# Robust nonfullerene solar cells approaching unity external quantum efficiency enabled by suppression of geminate recombination

**DOI:** 10.1038/s41467-018-04502-3

**Published:** 2018-05-25

**Authors:** Derya Baran, Nicola Gasparini, Andrew Wadsworth, Ching Hong Tan, Nimer Wehbe, Xin Song, Zeinab Hamid, Weimin Zhang, Marios Neophytou, Thomas Kirchartz, Christoph J. Brabec, James R. Durrant, Iain McCulloch

**Affiliations:** 1 King Abdullah University of Science and Technology (KAUST), KAUST Solar Center (KSC), Physical Sciences and Engineering Division (PSE), Thuwal, 23955-6900 Saudi Arabia; 2 Institute of Materials for Electronics and Energy Technology (I-MEET), Friedrich-Alexander-University Erlangen-Nuremberg, Erlangen, Germany; 3 Department of Chemistry and Centre for Plastic Electronics, Imperial College London, London, SW7 2AZ UK; 4 King Abdullah University of Science and Technology (KAUST), Core Labs, Thuwal, 23955-6900 Saudi Arabia; 5 IEK5-Photovoltaics, Forschungszentrum Jülich, 52425 Jülich, Germany; 6 Faculty of Engineering and CENIDE, University of Duisburg-Essen, Carl-Benz-Straße 199, 47057 Duisburg, Germany; 7 ZAE Bayern, Immerwahrstraße 2, Erlangen, 91058 Germany; 8 SPECIFIC IKC, Swansea University, Baglan Bay Innovation Centre, Port Talbot, Swansea, SA12 7AX UK

## Abstract

Nonfullerene solar cells have increased their efficiencies up to 13%, yet quantum efficiencies are still limited to 80%. Here we report efficient nonfullerene solar cells with quantum efficiencies approaching unity. This is achieved with overlapping absorption bands of donor and acceptor that increases the photon absorption strength in the range from about 570 to 700 nm, thus, almost all incident photons are absorbed in the active layer. The charges generated are found to dissociate with negligible geminate recombination losses resulting in a short-circuit current density of 20 mA cm^−2^ along with open-circuit voltages >1 V, which is remarkable for a 1.6 eV bandgap system. Most importantly, the unique nano-morphology of the donor:acceptor blend results in a substantially improved stability under illumination. Understanding the efficient charge separation in nonfullerene acceptors can pave the way to robust and recombination-free organic solar cells.

## Abstract (web summary)

The nonfullerene-based small molecules start to attract more attention for solar cell research than the fullerene acceptors due to their wider tunability. Here Baran et al. demonstrate nonfullerene-based solar cells with high power conversion efficiency of 12% and quantum efficiencies approaching 100%.

## Introduction

Solution-processed organic solar cells have traditionally relied mostly on the strong photon absorption provided by molecularly designed small molecule and polymer donor materials^1^. In order to dissociate photo-generated excitons created on the donor molecules, an electron accepting molecule is required. Traditionally, high power conversion efficiencies (PCEs) that are coupled with notable external quantum efficiencies (EQEs) had only been obtained using fullerene-based acceptor molecules. While fullerene acceptors allow ultrafast charge separation and fairly efficient charge transport^2^, their optical absorption is poor in comparison to many polymers and other small molecules in the visible range. Therefore, there have always been efforts to replace fullerene acceptors with molecules, which combine the advantages of fullerenes with a higher absorption strength^3^. Only recently, efficient nonfullerene acceptor molecules (NFAs) have emerged that compete or even outperform fullerenes when mixed with high-efficiency donor molecules^4–8^. In addition to the strong absorption of light, the success of NFAs can be in part attributed to the tunability of their optical properties^9^. By attaching strongly electron-withdrawing groups to an electron-donating core, it is possible to achieve a reasonably narrow bandgap due to the push-pull effect^3,10–12^ while maintaining the planarity of the molecule. This will result in a large overlap between the highest occupied molecular orbital and lowest unoccupied molecular orbital (LUMO) of the acceptor^13–15^. This orbital overlap leads to a greater oscillator strength and therefore a stronger absorption for light. In addition to the substantially improved absorption, polymer:NFA solar cells reported to date have frequently exhibited impressive open-circuit voltages when compared to their absorption onset^16^. There has been a recent report showing exciton separation in the picosecond range enabling efficient solar cells up to 9.5%^17^. However, such polymer:NFA devices have typically shown relatively modest EQEs, attributed to geminate recombination losses most likely associated with the small energetic offset driving charge separation in these devices^16,18^. Thus, the ability to reduce these losses while maintaining a rather high voltage for EQEs >80% has therefore become imperative to improve efficiency in polymer:NFA systems.

PBDTTT-EFT, more commonly known as PCE10 (or PTB7-Th), has been used extensively with NFAs and to date efficiencies (*η*) up to 11% have been achieved^7,19^. Early examples of PBDTTT-EFT:NFA systems in organic solar cells were in all-polymer blends. Initially, these blends were only able to achieve modest efficiencies (3–5%) due to the sub-optimal morphologies resulting from the unfavorable mixing of two polymer components in a blend, resulting in large recombination losses, which was accompanied by low EQEs with a maxima around 50%^20,21^. The PCE of all-polymer systems were later improved by addressing these morphological issues, creating more intermixed bulk heterojunctions possessing more balanced charge-carrier mobilities in the blends, thereby suppressing recombination. As a result, these all-polymer blends were able to achieve an EQE maximum of 65–70%^22–24^. The development of small-molecule NFAs allowed further improvements in the PBDTTT-EFT:NFA systems due to the lack of entropic driving force of polymers for mixing with small molecules^7^. The improved morphologies due to favorable mixing and higher absorption coefficients were reflected in improved PCEs in devices using small-molecule NFAs and coincided with greater EQEs in devices (80%)^25–27^. Despite these advances, the maximum potential of PBDTTT-EFT solar cells have not been reached with *V*_oc_ values >1 V and EQEs >80% thus far.

In this work, we report PBDTTT-EFT devices with an indacenodithiophene derivative NFA (EHIDTBR) that are able to achieve efficiencies of 12%, with a *V*_oc_ >1 V and EQE values around 90%. In the cells with the highest photocurrent, we achieve *J*_sc_ of 20 mA cm^−2^ (*J*_sc_ of 18.5 mA cm^−2^ in the cells with the highest efficiencies), which is exceptionally high for organic solar cells with an absorption onset around 1.6 eV. We elucidate the origin of this high *J*_sc_ and EQE with the findings that fast and efficient exciton separation smaller than 10 ps and >90% photoluminescence quenching yield followed by an efficient polaron formation with minimal geminate recombination losses. Moreover, depth profile analyses of the active layer of solar cell devices reveal that the strongly miscible character of PBDTTT-EFT and EHIDTBR leads to the formation of a robust microstructure and stable devices under illumination. In contrast, in the case of the fullerene devices, phase separation and demixing were evident over time causing reduced fill factor values even after 24 h. This study directly shows the suppression of geminate recombination for an NFA-based system combined with near unity quantum efficiency, exciton dissociation, and a robust morphology without vertical phase separation, yielding highly efficient (12%) and stable organic photovoltaic cells suitable for scale-up and commercial application.

## Results

### Photovoltaic characterization

**Fig. 1:**
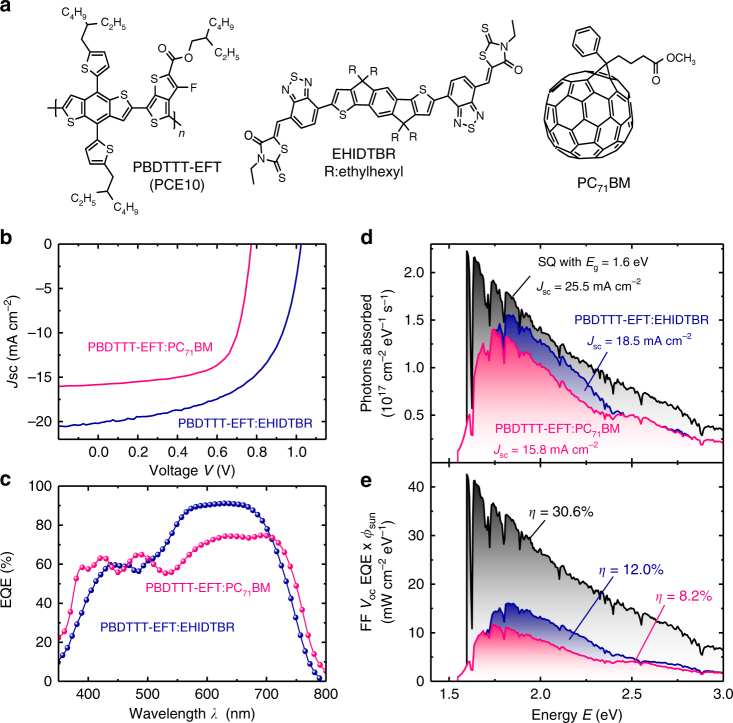
Chemical structures and photovoltaic characteristics of PBDTTT-EFT devices. **a** Chemical structures of PBDTTT-EFT, EHIDTBR, and PC_71_BM. **b** Current–voltage characteristics of PBDTTT-EFT:EHIDTBR and PBDTTT-EFT:PC_71_BM devices under 100 mW cm^−2^ illumination. **c** Internal and external quantum efficiency spectra of the corresponding devices. **d** External quantum efficiency multiplied with the solar spectrum for the data from **c** and for a step-function-like quantum efficiency (Shockley–Queisser limit) for a bandgap of 1.6 eV. **e** Product of solar spectrum, quantum efficiency, fill factor, and open-circuit voltage for the three cases from **d**. The area under the curves in **e** corresponds to a power density and is directly proportional to the efficiency of the devices

The photovoltaic properties of the solar cells are investigated by constructing an indium tin oxide (ITO)/electron-transport layer (ETL)/active layer (*d* of 80–120 nm)/MoO_3_/Ag architecture using different ETL modifications based on sol-gel ZnO and/or polyethylenimine (PEIE) for device optimization. Chemical structures of donor and acceptor molecules and current–voltage (*J*–*V*) characteristics of the PBDTTT-EFT:EHIDTBR (24 mg ml^−1^ in chlorobenzene solution) devices made using different ETLs in comparison to PBDTTT-EFT:PC_71_BM with 3% 1,8-diiodooctane (DIO) devices are shown in Fig. 1a, b, respectively. The key photovoltaic parameters for different ETL modifications in PBDTTT-EFT:EHIDTBR devices using ZnO, solvent modified ZnO (with 1% ethanolamine), and PEIE are illustrated in Table 1. In order to evaluate the performance of EHIDTBR in comparison to fullerenes, PBDTTT-EFT:PC_71_BM devices are constructed following previous reports with DIO (3%)^28,29^. The substantially higher *V*_oc_ of the nonfullerene devices results from tailored energy-level alignment between the frontier molecular orbitals at the donor/acceptor interface of the PBDTTT-EFT:EHIDTBR blend. The photovoltaic devices from PBDTTT-EFT:EHIDTBR blend exhibit an open-circuit voltage (*V*_oc_) of 1.03 V owing to the higher electron affinity (EA) (Supplementary Fig. 1) values of EHIDTBR (3.90 eV) compared to PC_71_BM (4.1 eV)^7,30^, where *V*_oc_ was limited to 0.78 V. In addition, a substantial improvement in the short-circuit current (*J*_sc_) up to 20 mAcm^−2^, an EQE maximum of 90% and fill factor (FF) of 0.63 values achieved yielding a PCE of 12% for PBDTTT-EFT:EHIDTBR devices without any thermal or solvent treatments. There has been a study about nonfullerene acceptor with PCEs up to 11%^19^, by far the highest efficiency reported using PBDTTT-EFT as the donor. However, the origin of the improved performance compared to fullerenes is not well understood and EQE >80% has not yet been achieved. The remarkably high *J*_sc_ and EQE of PBDTTT-EFT:EHIDTBR devices are investigated in detail in the following sections.

**Table 1:** Key photovoltaic parameters of PBDTTT-EFT:PC_71_BM and PBDTTT-EFT:EHIDTBR solar cell devices using different modified electron-transport layers

PBDTTT-EFT:EHIDTBR	*V*_oc_ (V)	*J*_sc_ (mA cm^−2^)	FF	*η*_max_(%) (average %)
ZnO	1.01 (±0.01)	18.2 (±0.4)	0.56 (±0.02)	10.5 (10.3)
ZnO + PEIE	1.02 (±0.01)	18.2 (±0.4)	0.56 (±0.02)	10.6 (10.3)
PEIE	1.03 (±0.01)	17.8 (±0.5)	0.63 (±0.01)	11.5 (11.3)
**ZnO** + **PEIE** + **EA** **(80** **nm)**	**1.03** **(±0.01)**	**18.5 (±0.3)**	**0.63 (±0.01)**	**12.0 (11.8)**
ZnO + PEIE + EA (150 nm)	1.03 (±0.01)	20.2 (±0.3)	0.57 (±0.02)	11.8 (11.5)
**PBDTTT-EFT:PC**_**71**_ **BM (3% DIO)**				
**ZnO**	**0.78 (±0.01)**	**15.9 (±0.4)**	**0.66 (±0.02)**	**8.5 (8.3)**

The lines that are highlighted in bold are the devices shown in Fig. 1

### Origin of high photocurrent and external quantum efficiency

Figure 1c shows the EQEs of the PBDTTT-EFT:EHIDTBR (modified with PEIE and EA, 80 nm) and the PBDTTT-EFT:PC_71_BM devices (90 nm). Figure 1d shows the product of EQE and the Air Mass (AM)1.5G solar spectrum for the two EQEs determined experimentally compared to the EQE_SQ_ in the Shockley–Queisser limit for a bandgap (*E*_g_) of 1.6 eV (EQE_SQ_ = 1 for *E* > *E*_g_ and 0 for *E* < *E*_g_). The area under the curves correlates with *J*_sc_. The PBDTTT-EFT:EHIDTBR cell achieves its high peak EQE by combining the absorption of donor and acceptor in the same photon energy region between 1.7 and 2.5 eV. Here, the PBDTTT-EFT:EHIDTBR-based device collects substantially more photons than the cell with PC_71_BM. The loss in EQE at higher photon energies is less relevant because of the reduced photon flux in the solar spectrum for energies above 2.5 eV. Figure 1e combines the data in 1d with the fill factor FF and the *V*_oc_ values to achieve PCE as a function of energy. The area under the curves in Fig. 1e is now directly proportional to the power density at the maximum power point and therefore also solar cell efficiency. While the PBDTTT-EFT:EHIDTBR device is still superior relative to the fullerene-based device, now the relatively low FF (63%) leads to substantial losses relative to the Shockley–Queisser limit, which has a FF around 90%. This clearly highlights that there is room for improvement for FF values, ideally for thicker devices to have even steeper absorption onsets that will boost EQEs in these systems.

**Fig. 2:**
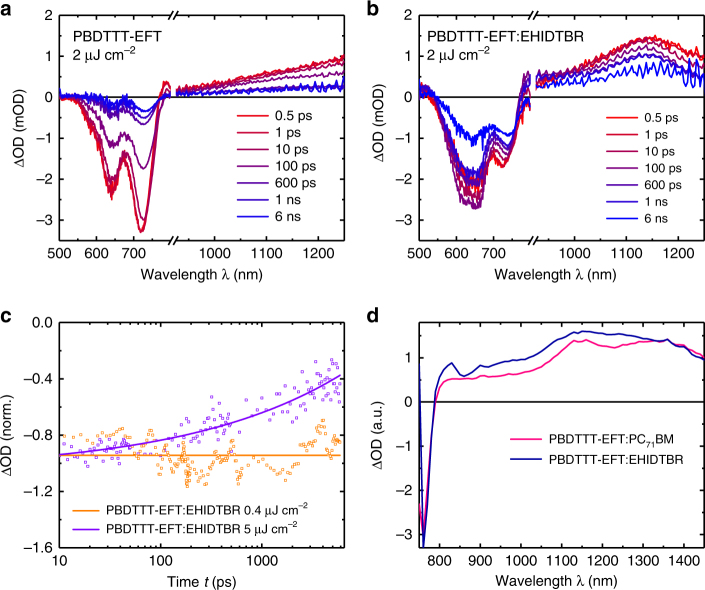
Spectroscopy data showing fast exciton dissociation and efficient polaron formation. Transient absorption spectra for a (**a**) neat PBDTTT-EFT film and (**b**) PBDTTT-EFT:EHIDTBR blend film, following excitation of the PBDTTT-EFT at 500 nm. Spectra are shown as a function of time delay after excitation from 0.5 ps to 6 ns. **c** The blend decay dynamics at 730 nm as a function of excitation density. At low excitation densities (0.4 µJ cm^−2^), no decay is apparent, indicative of an absence of geminate recombination dynamics; at higher excitation densities (5 µJ cm^−2^), significant decay is observed on this timescale (up to 6 ns) indicative of bimolecular, non-geminate recombination losses. These non-geminate recombination losses are also apparent as an absorbance loss at long time delays in **b** (measured at 2 µJ cm^−2^). **d** Photo-induced absorption spectra of PBDTTT-EFT:PC_71_BM and PBDTTT-EFT:EHIDTBR excited at 532 nm and recorded under vacuum of 10^−6^ Torr and a temperature *T* = 10 K illustrating the polaron yield of PBDTTT-EFT:EHIDTBR compared to PBDTTT-EFT:PC_71_BM

Transient dynamics of the photo-generated excitons and polarons provide information on the losses of an active layer, which then can be correlated to the photocurrent generation^31–34^. Figure 2 shows *fs*-transient absorption spectra (TAS) for neat PBDTTT-EFT and blended PBDTTT-EFT:EHIDTBR films, employing 500 nm excitation to selectively excite the polymer in both films. Control data for EHIDTBR and PBDTTT-EFT:PC_71_BM are shown in Supplementary Fig. 1. Blending PBDTTT-EFT with EHIDTBR or PC_71_BM results in the appearance of a long-lived, narrow photo-induced absorption band centered at 1150 nm, assigned to polymer polaron absorption. The shape of this photo-induced absorption is clearly distinct from that of either PBDTTT-EFT or EHIDTBR excitons (both of which exhibit steadily increasing absorption from 850 to 1100 nm, in contrast to the blend spectra). This polaron absorption appears for both blends from early times, with its spectral evolution being completed within 10 ps, indicating ultrafast charge separation on this timescale or faster (smaller than 10 ps). This suggests a blend morphology comprising primarily highly intermixed donor and acceptor molecules, enabling rapid exciton separation without requiring significant exciton diffusion. Photoluminescence quenching results supported these observations with strong photoluminescence (PL) quenching of EHIDTBR (99%) and PBDTTT-EFT emission (91%) in the blend relative to the neat films, indicative of efficient separation of both excitons (Supplementary Fig. 2). The lower photoluminescence quenching (PLQ) for PBDTTT-EFT excitons most probably arises from the presence of a small fraction of pure polymer in domains with diameters similar to or greater than the exciton diffusion length observed also in X-ray diffraction (XRD) traces (Supplementary Fig. 3a). Time-resolved luminescence decay of the neat and blend films are in agreement with the PLQ data showing 91% quenching when EHIDTBR is mixed with PBDTTT-EFT (1:1 w-w) (Supplementary Fig. 2i). Differential scanning calorimeter (DSC) traces reveal that neat EHIDTBR exhibit broad endothermic transitions at temperatures around 220 °C, attributed to the crystalline phase melt (Supplementary Fig. 3b). In contrast to EHIDTBR, no such thermally induced crystallization occurs during the heating cycle of PBDTTT-EFT due to its amorphous nature. The EHIDTBR crystalline transition in PBDTTT-EFT:EHIDTBR shows decreased enthalpy and minimized melting point depression. This implies that PBDTT-EFT partly diffuses into the EHIDTBR phase in the blend (Supplementary Fig. 3c). The cooling scan shows a reduced re-crystallization, which can be described as having a mixed PBDTTT-EFT:EHIDTBR region, comprising a crystalline acceptor phase and pure polymer domains, which reduces the PL quenching as well (Supplementary Fig. 2c, d).

It is also apparent from Fig. 2b at longer time delays (10 ps to 6 ns), the photo-induced absorption decays in amplitude, assigned to charge recombination losses. However, this was observed to be only present at high excitation densities, and was absent (within our signal to noise of ±10%) at low excitation densities (0.4 µJ cm^−2^) (Fig. 2c). We thus attribute the recombination losses observed at high laser intensities to non-geminate recombination and conclude that geminate (monomolecular) recombination losses are minimized in PBDTTT-EFT:EHIDTBR blend, at least over the timescale studied (we note these non-geminate losses were only observed under high laser excitation conditions, and are therefore not relevant to device performance under solar irradiance). Based on these results, it is clear that polymer exciton separation leads to almost complete charge dissociation, with a negligible (<10%) yield of geminate recombination from any bound “charge transfer” or “polaron pair” states formed at the donor/acceptor interface. The absence of geminate recombination losses combined with efficient exciton separation is likely to be a key reason behind the remarkably high *J*_sc_ and IQE (EQE/absorptance) data we report herein for PBDTTT-EFT:EHIDTBR solar cells (Supplementary Fig. 2e–h). The average IQE for the PBDTTT-EFT:EHIDTBR blend is above 95% throughout the visible region between 550 and 700 nm. The absence of geminate recombination losses in these devices, in contrast to, for example, the PffBT4T-2DT:FBR devices we have reported previously^16^, most probably results from the larger energy offsets driving exciton separation in the PBDTTT-EFT:EHIDTBR blend system (0.05 versus 0.25 eV, respectively.) To further elucidate the reasons for the enhanced *J*_sc_ of PBDTTT-EFT:EHIDTBR blend compared to PBDTTT-EFT:PC_71_BM, we used photo-induced absorption spectroscopy (PIA)^35^. By employing this technique, we can directly measure the concentration of long-lived photo-generated charge carriers in the blend^36^. Figure 2d depicts the PIA traces of PBDTTT-EFT blended with the two acceptors at 10 K. Notably, in agreement with the higher *J*_sc_ obtained for EHIDTBR-based solar cells, we found that the absorption feature at 1150 nm is considerably higher for the PBDTTT-EFT:EHIDTBR blend indicating larger polaron yield and/or longer polaron lifetimes compared to the PC_71_BM blend.

**Fig. 3:**
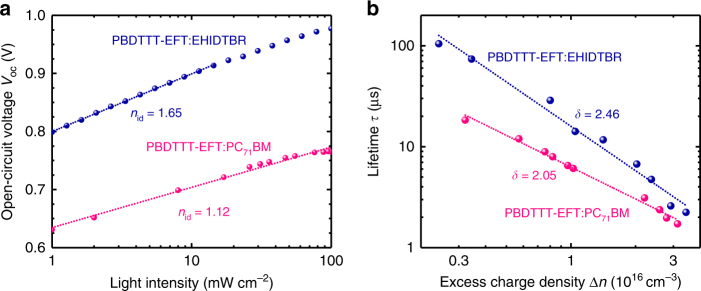
Analysis of recombination mechanism and dynamics in PBDTTT-EFT-based solar cells. **a** *V*_oc_ versus light intensity for PBDTTT-EFT:EH-IDTBR and PBDTTT-EFT:PC_71_BM devices. **b** Charge-carrier lifetime *τ*, obtained from TPV, as a function of charge density *n*, calculated from CE measurements under *V*_oc_ conditions (from 0.2 to 2 suns). The slope presents the recombination order (*δ*) of the studied systems. The dashed lines represent linear fits of the data

Taking into account the photovoltaic parameter evolution of PBDTTT-EFT:PC_71_BM devices compared to PBDTTT-EFT:EHIDTBR cells from Table 1, it is clear that the limiting factor for PBDTTT-EFT:EHIDTBR device efficiencies beyond 12% is the fill factor values around 0.63. The FF can be expressed as the compromise between recombination and extraction of charge carriers^37^. As aforementioned, PBDTTT-EFT:EHIDTBR blend lacks geminate recombination, thus we focus on gaining insight into non-geminate recombination from solar cell devices. We first investigate the photo-induced charge-carrier mobility (*μ*) of the devices by employing photo-induced charge-carrier extraction by linearly increasing voltage (photo-CELIV). The photocurrent traces of PBDTTT-EFT:PC_71_BM and PBDTTT-EFT:EHIDTBR solar cells (Supplementary Fig. 4) clearly reveal that the *t*_max_ has nearly identical for the two devices (*μ* of 2.1 × 10^−4^ cm^2^ V^−1^ s^−1^ and 2.0 × 10^−4^ cm^2^ V^−1^ s^−1^ for PBDTTT-EFT:PC_71_BM and PBDTTT-EFT:EHIDTBR devices, respectively). The closely similar charge-carrier mobility suggests that this is not the cause of the lower FF for the PBDTTT-EFT:EHIDTBR devices. As such, we carried out additional current–voltage characteristics and transient measurements for the analysis of recombination and lifetime of charge carriers in PBDTTT-EFT:EHIDTBR solar cells. An initial test of the dominant recombination mechanism in a solar cell already follows from dependence of the *V*_oc_ on the light intensity, *Φ*_in_^38^. One can express the *V*_oc_ as *V*_oc_ = *n*_id_ *kT*/*q*ln(*Φ*_in_) + const, where *n*_id_ is the ideality factor and *kT*/*q* is the thermal voltage. Values of *n*_id_ of 1 in a device with a fully depleted absorber can be assigned either to surface recombination or to direct recombination between electrons and holes, while *n*_id_ >1 indicates localized states in the bandgap being involved in recombination. As depicted in Fig. 3a, we obtain ideality factors of 1.15 and 1.65 for the PBDTTT-EFT:PC_71_BM and PBDTTT-EFT:EHIDTBR devices, respectively. This suggests that shallow trap states are involved in recombination in case of the EHIDTBR-based solar cells, which likely contributes to the decreased FF and is consistent with higher *n*_id_ obtained in other low-FF NFA-based devices^16^.

In parallel, we investigated the recombination mechanisms in more detail. As such, the methods transient photovoltage (TPV) and charge extraction (CE) has been studied to calculate the charge-carrier lifetime (*τ*) and excess charge-carrier density (Δ*n*)), respectively. Figure 3b shows the TPV lifetime as a function of the charge-carrier density that was extracted at the same light intensity, when switching from open circuit to short circuit while switching off the bias light at the same time. At a given extracted charge density Δ*n*, the PBDTTT-EFT:EHIDTBR-based solar cell has a higher lifetime, which contributes to the enhanced open-circuit voltage of the device. From the slope of lifetime or recombination rate *R* versus charge-carrier density, we can determine the reaction order *δ*, defined as the exponent in the relation *R* ~Δ*n*^δ^. For the PC_71_BM-based solar cell, the reaction order is close to 2 and the charge density scales with voltage with Δ*n* ~exp(*qV*_oc_/2*kT*) (Supplementary Fig. 5). NFA-based device has both higher ideality factors and higher reaction orders. In addition, charge density scales approximately with Δ*n* ~ exp(*qV*_oc_/3.3*kT*) (Supplementary Fig. 5), which also suggests the existence of localized shallow trap states in the bandgap^39^. It appears that the PBDTTT-EFT:EHIDTBR device offers more degrees of freedom and also more energetic disorder, which could limit the FF values to 65%^39^.

### Influence of phase separation on lifetime and degradation

**Fig. 4:**
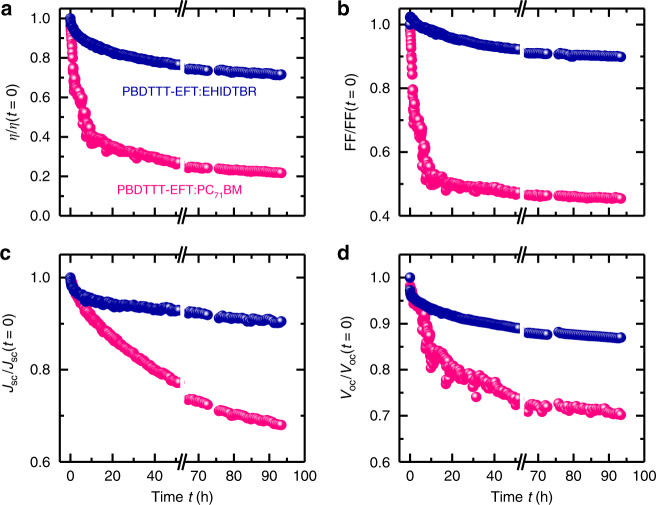
Stability data for fullerene and nonfullerene devices. Time evolution of device performance and figures of merit of PBDTTT-EFT:EHIDTBR and PBDTTT-EFT:PC_71_BM solar cell devices under illumination of metal halide lamp in N_2_ atmosphere

**Fig. 5:**
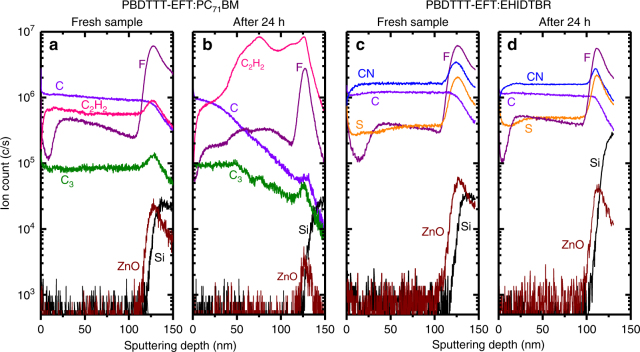
Depth profiles before and after degradation. Negative SIMS depth profiling of PBDTTT-EFT:PC_71_BM (**a**, **b**) and PBDTTT-EFT:EHIDTBR (**c**, **d**) blends prior to light radiation and subjected to light radiation equivalent to 24 h. 0 nm sputtering depth represents the top surface and 150 nm shows the substrate (ZnO)

A robust nano-morphology against irradiation is required for long lifetime solar cells. There have been efforts for such a nano-morphology in Organic solar cells (OSCs) using molecular locks or alloy acceptors in fullerene solar cells^40,41^; however, the high-efficiency devices usually suffered from optimization using solvent additives that cause demixing of the polymer:fullerene blend^42–44^. Recently, NFA devices outperformed fullerene device stabilities with interface engineering using low-temperature TiO_*x*_ for conventional structures^45,46^; nevertheless a recipe for a robust nano-morphology of the polymer-NFA blend is missing and has not been explored yet. To study the severity of degradation, both PBDTTT-EFT:PC_71_BM and PBDTTT-EFT:EHIDTBR devices were in an environmentally controlled chamber (O_2_ and H_2_O lower than 1 p.p.m.) and the photovoltaic parameters were probed simultaneously under a metal halide lamp (irradiated at 100 mW cm^−2^). Figure 4 summarizes the time evolution of photovoltaic parameters of both devices for up to 100 h of degradation. PC_71_BM-based PBDTTT-EFT devices suffer from an abrupt decrease in FF and thus efficiency even after 24 h, whereas there is only a gradual decrease in efficiency (around 20%) for the EHIDTBR device over a period of 100 h, which is similar to other NFA systems^7,13,47^. Generally, for commercial application, ultraviolet (UV) filter can be adopted to avoid the negative impact of UV light on the device stability. Thus, we replaced the metal halide lamp (MHI) lamps white LED lights^48^. As a result, PBDTTT-EFT:EHIDTBR cells show high stability (PCE drop <10%) up to 1000 h (Supplementary Fig. 7), which is a benchmark for long-term stability. Dynamic secondary ion mass spectrometry (SIMS) was used to understand the depth profile of both blends and explain the huge difference in stability with NFA devices. The advantage of SIMS relies on its capability to track the elemental distribution in depth allowing us to obtain valuable information regarding the homogeneity and miscibility of the donor and acceptor across the active layer^28,49^. The SIMS data in Fig. 5 illustrate the distribution of elements and ions as a function of depth within the PBDTTT-EFT:PC_71_BM and PBDTTT-EFT:EHIDTBR blends spin-cast on zinc oxide(ZnO)/Si substrates. We compared SIMS data obtained from fresh samples (prior to light radiation) to those acquired for samples subjected to metal halide light radiation (equivalent to 24 h). In order to identify the signature of each component in both blends, the fluorine element described by the F^−^ signal is ascribed to PBDTTT-EFT, whereas the EHIDTBR is tracked by following the CN^−^ signal. Although the PC_71_BM is composed of C, O, and H, which are also present in the PBDTTT-EFT polymer, its behavior may be examined by following C^−^ and C_3_^−^ signals as will be discussed below. Assuming the aforementioned assignments, the SIMS data shows clearly that the PBDTTT-EFT:EHIDTBR blend has a very similar depth profile before and after light radiation. The elemental depth distribution of the F^−^ and CN^−^ signals indicates a homogeneous distribution of both PBDTTT-EFT and EHIDTBR across the active layer. It should be noted that the increase of all ion signals at the ZnO interface is an artefact due to a change of the chemical environment. In contrast to PBDTTT-EFT:EHIDTBR, the PBDTTT-EFT:PC_71_BM mixture prepared with 3% DIO represents a pronounced vertical phase separation and demixing following the light radiation. These observations were also in line with the high-resolution transmission electron microscopy of the active layer blends. (Supplementary Fig. 6) PBDTTT-EFT, denoted by F^−^ and C_2_H_2_^−^ is clearly pushed toward the ZnO interface. However, C^−^ and C_3_^−^ signals are more concentrated in the first 50 nm depth beyond which they exhibit a clear decrease throughout the active layer, which is more likely to represent the fullerene acceptor. Thus fullerene acceptors are located closer to the surface than ZnO when the PBDTTT-EFT:PC_71_BM blend is subjected to light radiation due to demixing that results in severe reduction in FF values. We believe that this is due to high boiling point solvent additive (DIO) used to optimize the morphology of fullerene devices. A similar meta-stable morphology observation was reported by Li et al.^42^, where DIO had effect not only in long-term lifetime of the devices but also in the initial measurements as burn-in that causes spinodal demixing. These results suggest that high-efficiency solar cell devices optimized with solvent additives would not be the choice for long-term stable commercial applications.

In conclusion, we report a high-performance organic photovoltaics (OPV) system based on a small-molecule acceptor that delivers a PCE of 12% with an EQE around 90% and a *V*_oc_ of 1.03 V. We elucidate that the concomitant effect of reduced geminate recombination and efficient charge separation leads to an exceptionally high *J*_sc_ of 20 mA cm^−2^. In parallel to the opto-electronic analysis, we demonstrate that the NFA cells depict a more robust microstructure toward light degradation compared to the fullerene counterparts. Most obviously, the LUMO level offset between PBDTTT-EFT and EHIDTBR is still sufficient to drive efficient exciton separation and charge dissociation. It is also surprising that this blend consisting of an amorphous donor does not foster geminate losses but yields charges efficiently. One of the possible explanations is that, as illustrated from the DSC, TEM, PLQ, and XRD data, a highly intermixed region of PBDTTT-EFT and EHIDTBR ensures efficient exciton dissociation. The larger pure domains of PBDTTT-EFT and crystalline acceptor phase of EHIDTBR (from DSC) enables the spatial separation of photo-generated carriers. In addition, an efficient charge separation is enabled by sufficiently large energetic offset and suppress geminate recombination losses. Overcoming trap-assisted recombination remains a challenge for organic solar cells and an elegant approach to overcome this could be ternary blend, in which a more ordered polymer or small molecule is added to a binary system to facilitate charge transport, which has been shown to result in a higher FF^28,50^. Overall, these results suggest that overcoming trap-assisted recombination, i.e., reducing the defects, by tackling FF losses with further interface engineering creates the opportunity for durable OPV systems for commercialization.

## Methods

### Materials

PBDTTT-EFT was purchased from Cal-Os chemicals. EHIDTBR was synthesized according to the previous reports^13^. PC_71_BM (99.5%) was purchased from Solenne BV. All the other materials and solvents are received from Sigma-Aldrich and used without further purification.

### Device fabrication and characterization

The inverted architecture devices were processed in inert atmosphere using ITO/ETL/active layer/MoO_3_ (10 nm)/Ag (100 nm) stack, where ETL were zinc oxide (using sol-gel method) and ethoxylated PEIE, active layers were PBDTTT-EFT:EHIDTBR (1:2.5, w-w%) and PBDTTT-EFT:PC_71_BM (1:1,5, w-w%). Cleaning of the pre-structured ITO substrates has been done using deionized water, acetone, and isopropyl alcohol (10 min each). Eight minutes of plasma treatment was applied to treat the substrates. A thin layer of ZnO was deposited from a precursor solution of zinc acetatedihydrate (2 ml 2-methoxyethanol and 60 μL ethanolamine). A 30–40 nm ZnO layer was formed after 20–25 min annealing at 150 °C. The modification of ZnO layer was done with either spin-coating a 1% ethanolamine in 2-methoxyethanol on existing annealed ZnO layers followed by annealing at 100 °C for 10 min, spin-casting PEIE solution in ethanol using 4000 r.p.m., and annealing at 80 °C for 10 min or both procedures following the consecutive order on top of ZnO layer (ZnO + PEIE + EA). Active layers were spin-cast from 24 mg ml^−1^ concentrated solutions in chlorobenzene to result in 90–100 nm thick layers. Thick devices were prepared using 28 mg ml^−1^ total solution. MoO_3_ (10 nm) and silver (100 nm) were evaporated using a shadow mask (with an aperture of 0.045 cm^2^) under vacuum (1 × 10^−6^ mbar). No post annealing was performed to any of the devices. *J*–*V* characteristics were measured under dark and illumination using a xenon lamp from oriel Instruments (AM 1.5 G, 100 mW cm^−2^). EQE spectra were obtained using a silicon photodiode reference with 100 W Tungsten–Halogen lamp. Six pixels are produced for averaging the results.

### Photo-CELIV measurements

Photo-CELIV measurements were performed with a 405 nm laser-diode. An internal 50 Ω resistor is used with an oscilloscope (Agilen technologies DSO-X 2024A) to record the current transients. A fast electrical switch was used to isolate the cell and prevent CE or sweep out during the laser pulse and the delay time. After a variable delay time, a linear extraction ramp was applied via a function generator. The ramp, which was 60 μs long and 2 V in amplitude, was set to start with an offset matching the *V*_oc_ of the cell for each delay time.

### TPV and CE measurements

Solar cells were brought to *V*_oc_ condition with a 405 nm laser-diode. The light intensity is adjusted over a range of 0.2 to 2 suns (with less than 0.5% error) by controlling the waveform generator (Agilent 33500B) and the intensity of a linear photodiode. A second 405 nm laser is used to induce a small perturbation in the device by a function generator (Agilent). The short laser pulse intensity (50 ns) was used to adjust the voltage perturbation below 10 mV (about 5 mV). The voltage decays to initial steady state after the pulse in a single exponential decay. The times of the decay was determined using a linear fit to the log plot of the voltage transient and small perturbation charge-carrier lifetime. In CE studies, the 405 nm laser pulsed the solar cells for 200 μs to bring the devices to a constant open-circuit voltage condition. Once the illumination stops, an analog switch was triggered to bring the solar device to Jsc condition (50 Ω) with <50 ns.

### Transient absorption spectroscopy (*fs*-TAS)

TAS measurements were performed by first generating a 800 nm laser pulse (1 kHz, 90 fs) from a commercial Solstice (Newport Corporation) Ti:sapphire regenerative amplifier. A part of the laser pulse optically directed through a TOPAS-Prime (light conversion) optical parametric amplifier to generate a 500 nm excitation pump laser. The other part of the laser output was used to generate the probe light in visible/near-IR continuum by sapphire crystals (0.4 and 1 cm thickness, respectively). The spectra and decays were obtained by a HELIOS transient absorption spectrometer (500–1300 nm) to 6 ns. The samples were measured in N_2_ atmosphere.

### Photo-induced absorption measurements

Photo-induced absorption (PIA) studies were performed by exciting the sample with a 405 nm laser while simultaneously probing the sample with a white lamp. The PIA spectra of the sample were dispersed by a 1200 lines per mm grating monochromator (iHR320) and detected by a silicon detector through lock-in technique.

### Ageing and lifetime measurements

The photovoltaic devices are constructed in the same way described in the device characterization part. The moisture and O_2_ concentrations were kept below 0.5 p.p.m. for the ageing experiments in N_2_ atmosphere in a closed chamber system. Metal halide lamp with UV part is used to irradiate the samples (T: 30 to 33 °C) for 24 h and 1 week for long-term degradation.

### Dynamic secondary ion mass spectrometry

Depth-profiling experiments were performed on a dynamic SIMS instrument from Hiden analytical company (Warrington-UK) operated under ultra-high vacuum conditions, typically 10^−9^ torr. A continuous Ar^+^ beam of 2 keV energy was employed to sputter the surface while the selected ions were sequentially collected using a MAXIM spectrometer equipped with a quadrupole analyzer. In order to avoid the edge effect, the SIMS data were extracted from the acquisition area of 50 × 50 µm^2^ centered in the middle of the sputtered area estimated to be 500 × 500 µm^2^ using an adequate electronic gating. Assuming a constant sputtering rate, the conversion of timescale to depth scale was carried out by measuring the depth of the crater generated at the end of the depth-profiling experiment using a stylus profiler from Veeco.

### Transmission electron microscopy

Dark-field transmission electron microscopy (TEM) images of bulk heterojunction (BHJ) films for morphological characterization were performed with a FEI Titan 80–300 TEM equipped with an electron monochromator, a Gatan imaging filter (GIF) Quantum 966 and a Cs probe corrector. All images were obtained in scanning TEM mode at 80 kV. Films were deposited on a PEDOT:PSS sacrificial layer in the following configuration PEDOT:PSS/ZnO/BHJ and they were then emerged in deionized water. Floating films of ZnO/BHJ were collected with carbon-coated gold grids from Electron Microscopy Sciences. Films were then dried overnight in a vacuum chamber to remove any water residues.

### X-ray diffraction measurement

X-ray diffraction measurement (XRD) measurement was conducted on PBDTTT-EFT and EHIDTBR neat and blend films, which were prepared by drop-casting about 0.25 ml of solutions onto 2 cm × 1 cm substrates and left to dry overnight at room temperature. The XRD is equipped with a PANALYTICAL X’PERT-PRO MRD diffractometer using a nickel-filtered Cu K α1 beam and a X’CELERATOR detector, employing a current *I* = 40 mA and an accelerating voltage *U* = 40 kV.

### Differential scanning calorimetry

Differential scanning calorimetry (DSC) experiments were carried out with a TA Instruments DSC Q20 at a heating rate of 5 °C per min under nitrogen; heating (and cooling) cycles were recorded. Samples were prepared by drop-casting the materials from chlorobenzene solution onto glass slides and allowing the solvent to evaporate under ambient conditions. The dry films were then scrapped off and transferred into DSC pans.

### Time-resolved photoluminescence

Time-resolved photoluminescence (TRPL) spectra were recorded using a high-resolution streak camera system (Hamamatsu C10910) where the excitation beam is adjusted to 650 nm using Spectra Physics MaiTai eHP from a mode-locked Ti:sapphire laser (Coherent Mira 900D). During the measurements, emission of the samples was detected by a monochromator attached to a Universal Hamamatsu C6860 streak camera with a temporal resolution of 4 ps.

### Ellipsometry measurements

For ellipsometry measurements, PBDTTT-EFT:EH-IDTBR was spin coated onto silicon wafers coated with 25 nm of SiO_2_. The J.A. Woollam M2000 ellipsometer was used to collect the raw ψ and Δ values that describe polarization change over the visible range. The *n*, *k* values were obtained using Complete EASE software. Models were developed and fitted to the ψ and Δ data until a suitable fit was found, indicated by a mean squared error (MSE) value <2.

### Data availability

The data that support the findings of this study are available on request from the corresponding author (D.B.).

## Electronic supplementary material

Supplementary Information
