# Supplementary material for: Robust nonfullerene solar cells approaching unity external quantum efficiency enabled by suppression of geminate recombination
Source: Nat Commun. 2018 May 25;9:2059. doi: 10.1038/s41467-018-04502-3 (PMC5970237; doi:10.1038/s41467-018-04502-3)
Supplement: Supplementary file 1 — Supplementary Information [file 41467_2018_4502_MOESM1_ESM.pdf]

Robust nonfullerene solar cells approaching unity external quantum efficiency enabled by suppression of geminate recombination

Baran et al.

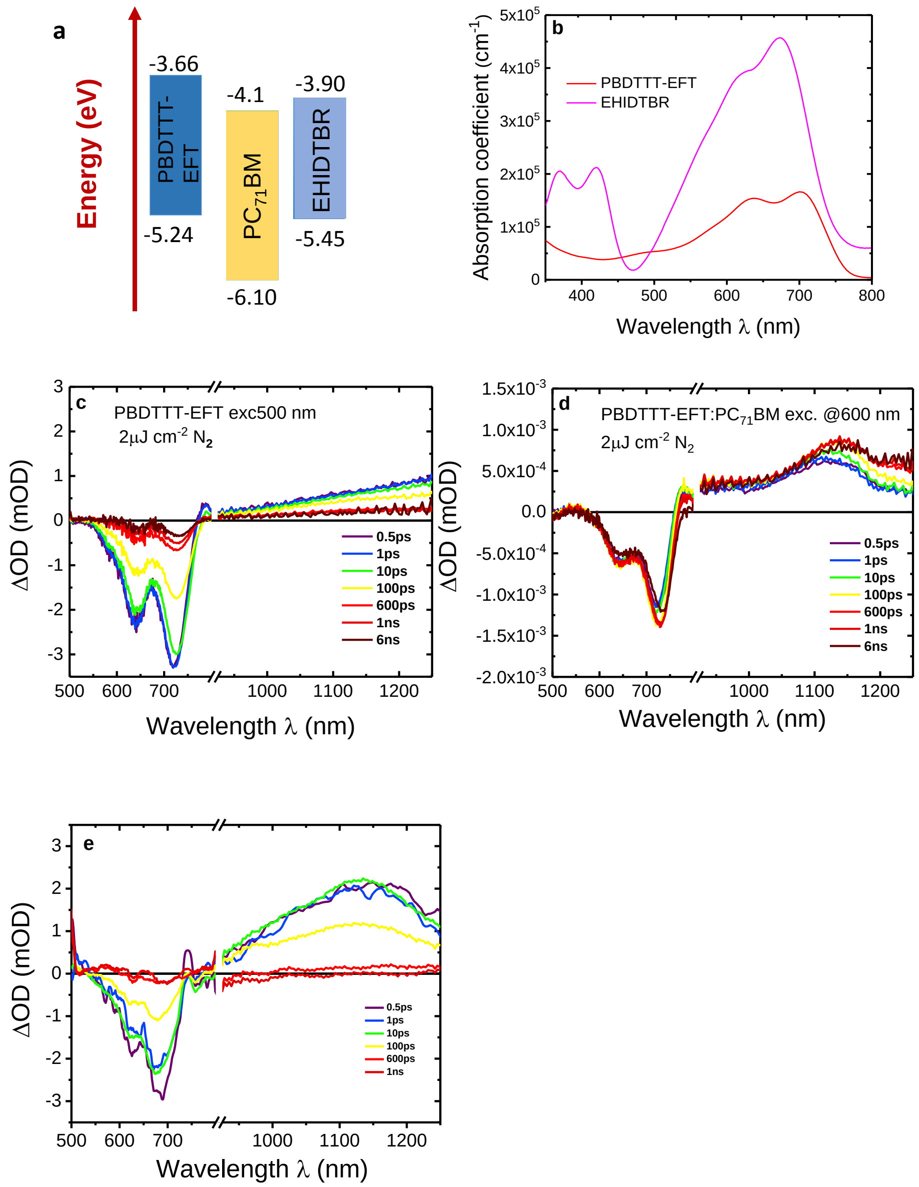

Supplementary Figure 1| **Energy diagrams and optical data of PBDTTT-EFT with fullerene and NFA**. a) Energy diagrams of donor and acceptor molecules. b) Absorption coefficient of neat PBDTTT-EFT and EHIDTBR films. Transient absorption spectra of c) a neat PBDTTT-EFT, d) a PBDTTT-EFT:$PC_{71}BM$ blend and e) a neat EH-IDTBR film employing the same excitation densities (2μJ cm^{-2}). Spectra are corrected for the number of photon absorbed at 500 nm and 600 nm excitation wavelengths and are shown as a function of time delay from 0.5 ps to 1 ns.

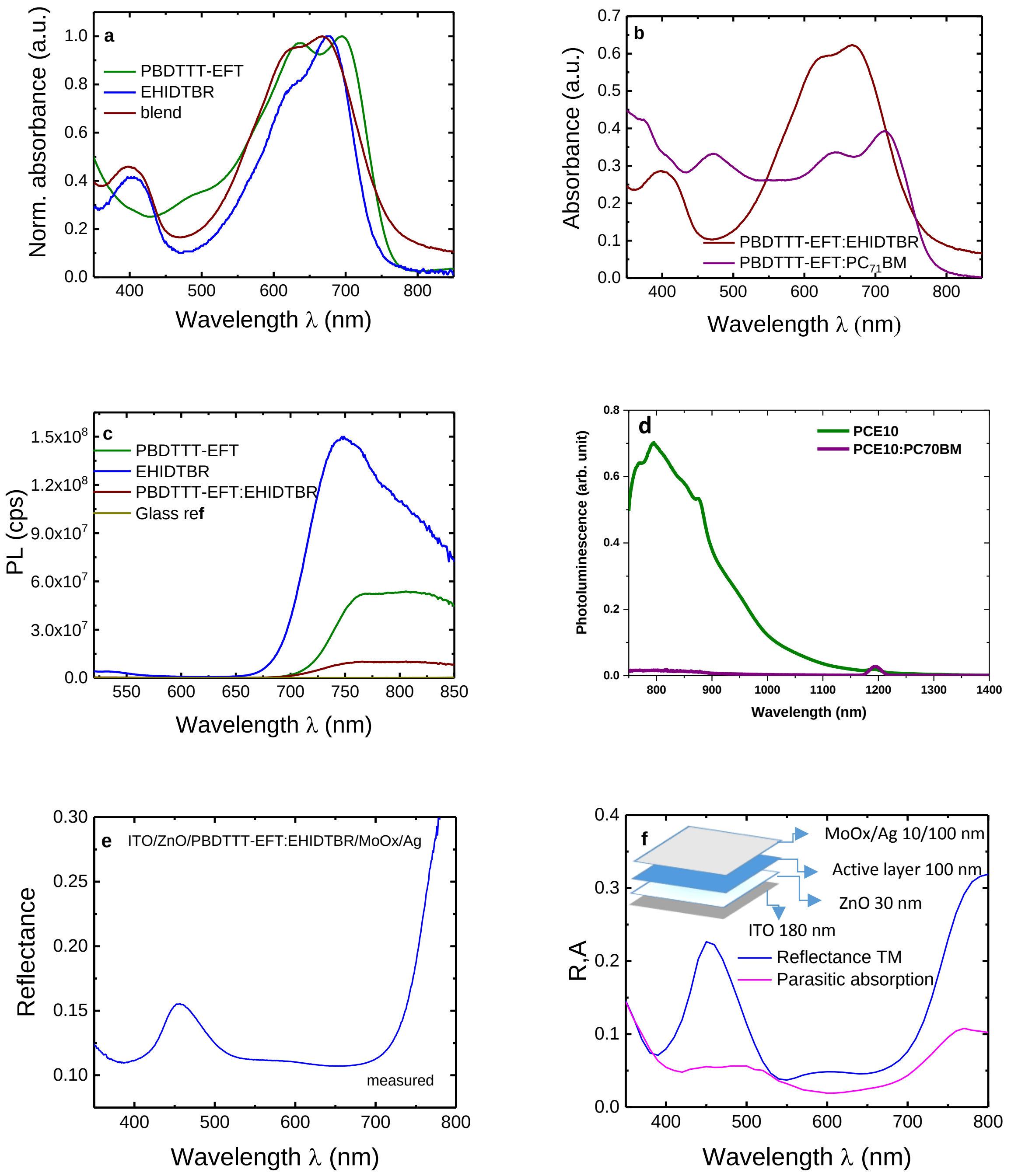
a
PBDTTT-EFT
EHIDTBR
blend
Norm. absorbance (a.u.)
Wavelength λ (nm)
b
PBDTTT-EFT:EHIDTBR
PBDTTT-EFT:PC71BM
Absorbance (a.u.)
Wavelength λ (nm)
c
PBDTTT-EFT
EHIDTBR
PBDTTT-EFT:EHIDTBR
Glass ref
PL (cps)
Wavelength λ (nm)
d
PCE10
PCE10:PC70BM
Photoluminescence (arb. unit)
Wavelength (nm)
e
ITO/ZnO/PBDTTT-EFT:EHIDTBR/MoOx/Ag
measured
Reflectance
Wavelength λ (nm)
f
MoOx/Ag 10/100 nm
Active layer 100 nm
ZnO 30 nm
ITO 180 nm
Reflectance TM
Parasitic absorption
R,A
Wavelength λ (nm)

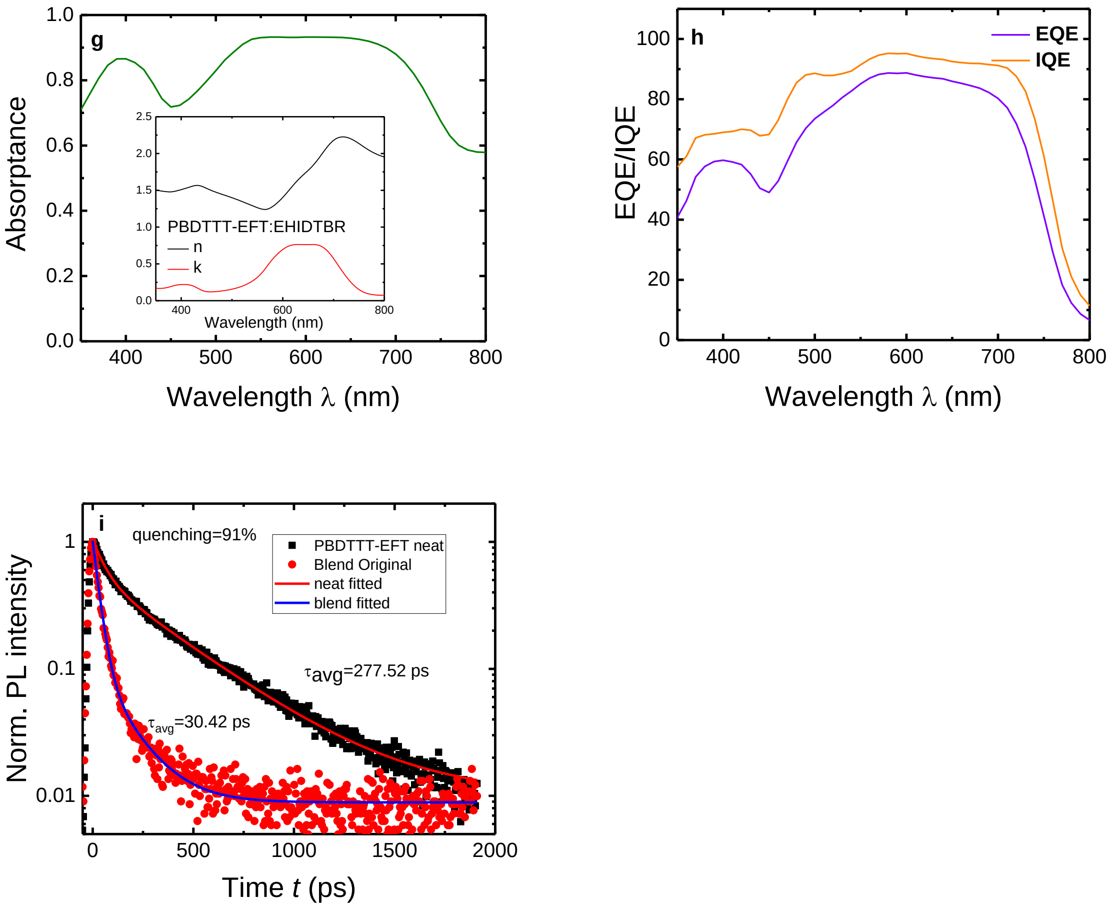

Supplementary Figure 2| **Absorptance and emission characteristics of donor and acceptor molecules.** a) Normalized absorbance spectra of neat materials and PBDTTT-EFT:EHIDTBR blend. b) Absorbance of PBDTTT-EFT:$PC_{71}BM$ and PBDTTT-EFT:EHIDTBR blends with similar thickness (approximately 90nm) showing absorption strength of EHIDTBR acceptor. c) Relative steady-state photoluminescence spectra of corresponding neat materials and PBDTTT-EFT:EHIDTBR blend showing 91% PL quenching of the blend as well as emission strength of EHIDTBR compared to PBDTTT-EFT. d) Relative steady-state photoluminescence spectra of corresponding neat material and PBDTTT-EFT: $PC_{71}BM$ blend showing 98% PL quenching. PL emission of neat and blend films are corrected for the number of photon absorbed. e) Absorption in a complete PBDTTT-EFT:EHIDTBR cell as measured using a traditional reflection-mode absorption measurement where only specular reflection is detected. f) Reflectance and parasitic absorption of the whole device stack calculated using a transfer matrix model with *nk* values obtained from ellipsometry (panel *g* inset) and the assumed layer thicknesses as indicated in the inset. g) Simulated absorptance of a complete PBDTTT-EFT:EHIDTBR solar cell based on the reflectance and parasitic absorption data from panel (f). Note that the *nk* data of the blend is not particularly accurate close to the optical gap. h) EQE and calculated IQE (EQE/Absorptance) graphs for PBDTTT-EFT:EHIDTBR using the simulated absorptance data from panel (g). The small difference (5-7%) between EQE and IQE is due to parasitic absorption losses of the interface and electrode layers. i) Time-resolved luminescence decay of neat PBDTTT-EFT and PBDTTT-EFT:EHIDTBR blend excited at 650nm.

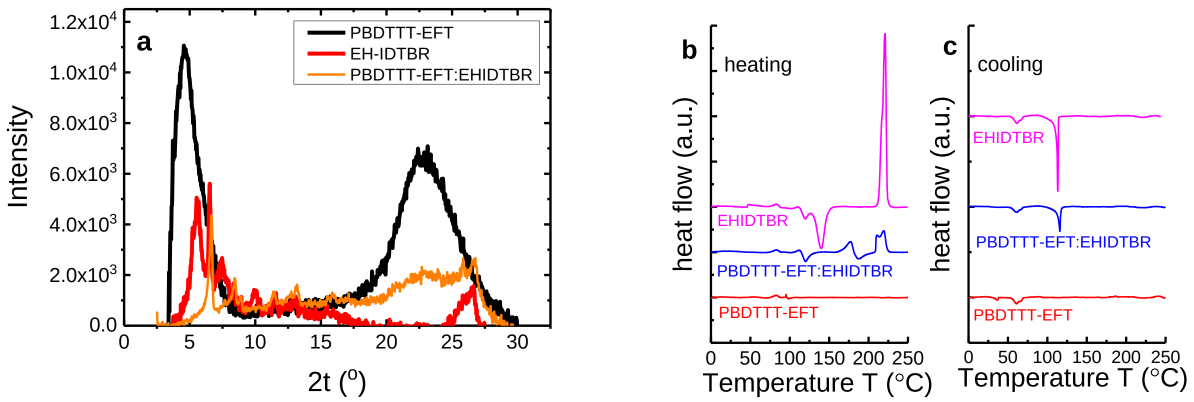

Supplementary Figure 3| **X-ray diffraction and DSC heating/cooling traces of neat and blend materials.** a) The blend peak at 2θ of around 23° corresponds to the PBDTTT-EFT π-π packing [1] while the peak at 4.6°correlates to the lateral packing diminishes in blend suggesting this packing is disrupted by the formation of an intermixed PBDTTT-EFT and EHIDTBR. The peaks at 6.5° and 27° correspond to EHIDTBR which has been previously shown to exhibit a face-on packing [2]. b and c) DSC profiles of heating/cooling scans of neat and binary blends of PBDTTT-EFT, EHIDTBR and PBDTTT-EFT:EHIDTBR.

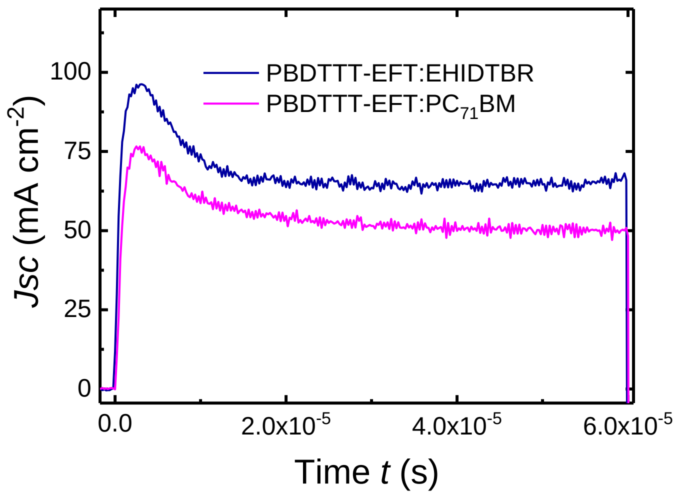

Supplementary Figure 4| **Photo-CELIV curves with laser excitation @405 nm.**

All the transient were recorded by applying a 2V per 60μs linearly increasing reverse bias pulse and a delay time (t_d) of 1 μs.

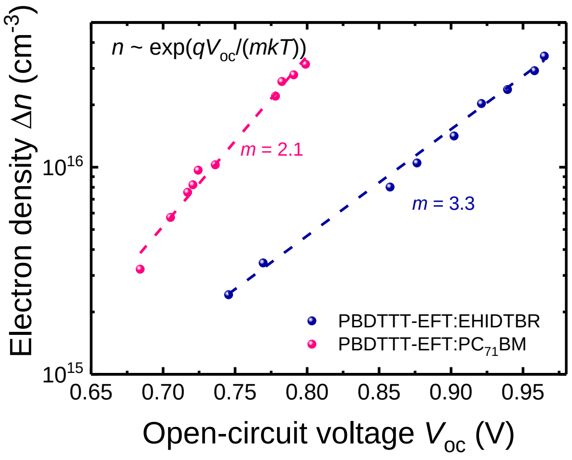

Supplementary Figure 5| **Electron density vs. open-circuit voltage data of PBDTTT-EFT:PC_{71}BM and PDTTT-EFT:EHIDTBR devices**. m is correlated to the energetic disorder.

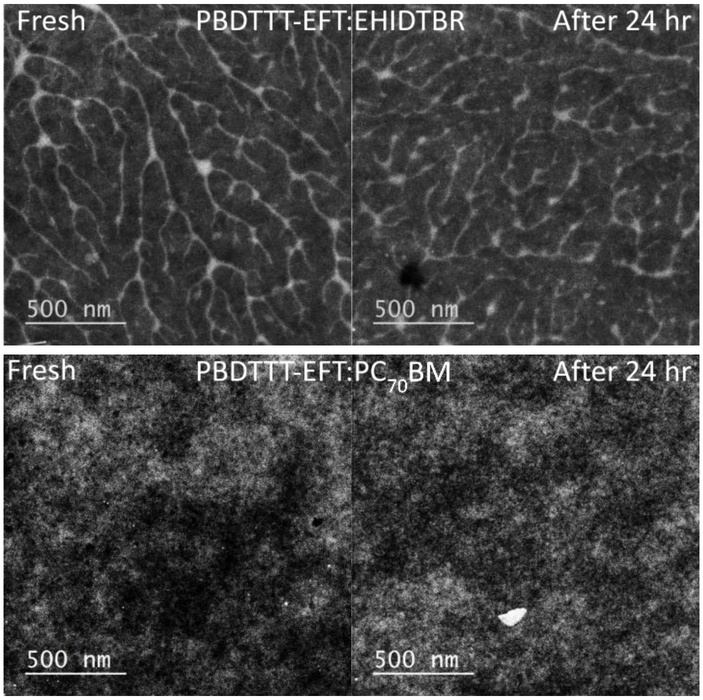

Supplementary Figure 6|**Transmission electron microscopy images if PBDTTT-EFT:EHIDTBR and PBDTTT-EFT:PC_{71}BM.** Blends before and 24h after irradiation with metal halide lamp T=between 30 and33 C. Light regions represent electron rich domains which is the acceptor EHIDTBR and PC_{71}BM in this case, respectively.

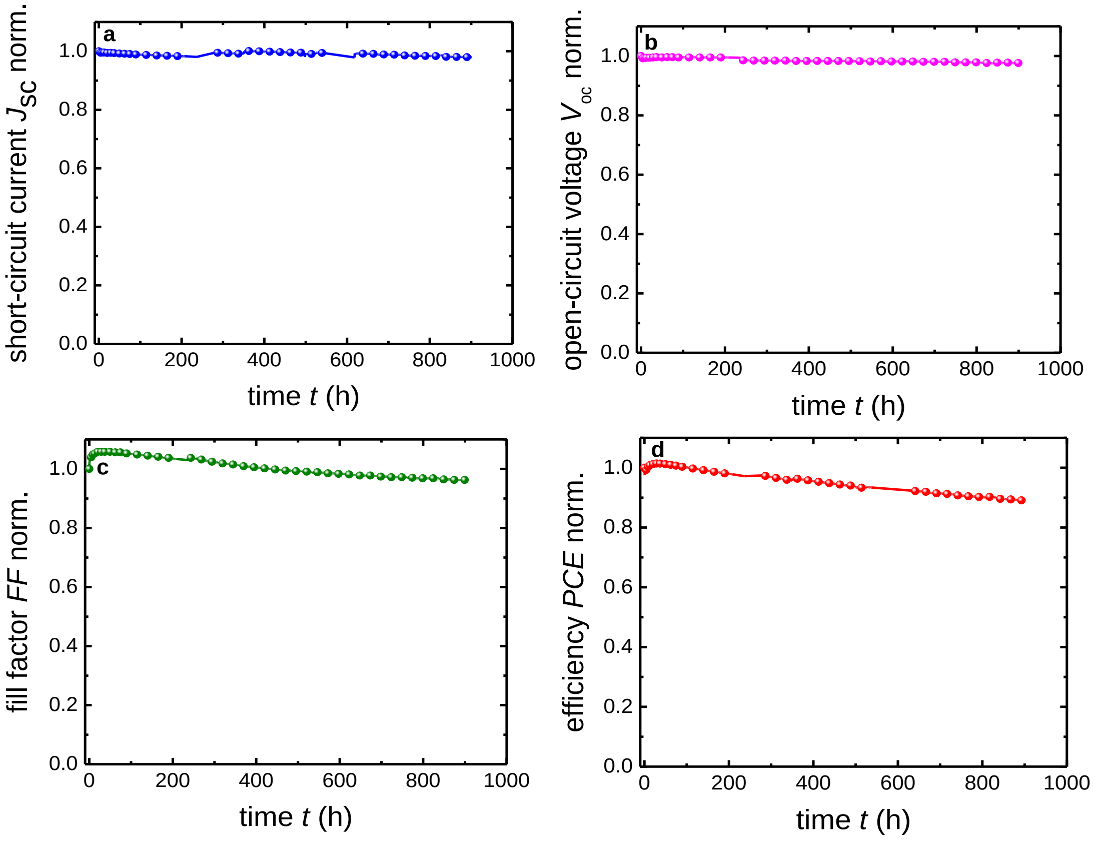

Supplementary Figure 7|**Time evolution of the key photovoltaic parameters**. PBDTTT-EFT:EHIDTBR devices (average of 6 devices) under 1000h of light exposure.(white LEDs are used to match 1 sun conditions.)[3]

Supplementary References:

1. Soon, Y. W., et. al. Adv. Funct. Mater., 2014, 24, 1474.
2. Holliday, S. et al. Nat. Commun., 2016, 7,11585.

3. Gasparini, N. *et al.* Adv. Energ. Mater., 2017, 7, 1700770.
